# Cross-Correlation of Motor Activity Signals from dc-Magnetoencephalography, Near-Infrared Spectroscopy, and Electromyography

**DOI:** 10.1155/2010/785279

**Published:** 2010-01-24

**Authors:** Tilmann H. Sander, Stefanie Leistner, Heidrun Wabnitz, Bruno-Marcel Mackert, Rainer Macdonald, Lutz Trahms

**Affiliations:** ^1^Division of Medical Physics, Physikalisch-Technische Bundesanstalt, Abbestraße 2-12, 10587 Berlin, Germany; ^2^Department of Neurology, Charité Campus Benjamin Franklin, 12200 Berlin, Germany; ^3^Department of Neurology, Vivantes Auguste Viktoria Klinikum, 12157 Berlin, Germany

## Abstract

Neuronal and vascular responses due to finger movements were synchronously measured using dc-magnetoencephalography (dcMEG) and time-resolved near-infrared spectroscopy (trNIRS). The finger movements were monitored with electromyography (EMG). Cortical responses related to the finger movement sequence were extracted by independent component analysis from both the dcMEG and the trNIRS data. The temporal relations between EMG rate, dcMEG, and trNIRS responses were assessed pairwise using the cross-correlation function (CCF), which does not require epoch averaging. A positive lag on a scale of seconds was found for the maximum of the CCF between dcMEG and trNIRS. A zero lag is observed for the CCF between dcMEG and EMG. Additionally this CCF exhibits oscillations at the frequency of individual finger movements. These findings show that the dcMEG with a bandwidth up to 8 Hz records both slow and faster neuronal responses, whereas the vascular response is confirmed to change on a scale of seconds.

## 1. Introduction

The methodologies to characterize neurovascular coupling in humans [1] can be separated at least into two categories. The first relies on two sequential measurements of the same subject and infers coupling parameters, the second performs multimodal synchronous measurements of neuronal and vascular effects, and the coupling is observed directly. The second methodology is technically more complicated, but the effect of a subject's performance changing between two measurements is eliminated. Furthermore it allows to study the coupling on continuous time series, that is, without relying on epoch averages removing the variability between individual epochs. Results from these approaches complement the detailed findings for neurovascular coupling obtained from invasive studies in animals [2].

One possibility to study neurovascular coupling by synchronous measurements was described in [3–5] combining dc-magnetoencephalography (dcMEG) with time-resolved near-infrared spectroscopy (trNIRS) during intermittent finger movements. These synchronous measurements were so far limited to a bandwidth from DC to 0.4 Hz due to the modulation technique used for the dcMEG [4]. With the possibility to obtain unmodulated dcMEG in a magnetically extremely shielded room [6, 7] the bandwidth of the synchronous measurements is considerably increased; that is, slow signal changes close to DC and standard neuronal responses above 1 Hz can be recorded at the same time. To keep in line with earlier literature the term dcMEG is maintained, but it denotes here a bandwidth of the signal from 0.01 Hz to about 8 Hz. Oscillatory signals at higher frequencies such as the *γ*-rhythm will not be considered here. 

An alternative method [8] used trains of somatosensory MEG responses at latencies between 20 and 100 milliseconds and the related NIRS responses. These fast MEG responses can be measured in standard shielded rooms, where the lower MEG bandwidth limit is 0.1 Hz. The fast neuronal responses are well suited to investigate the linearity of neurovascular coupling, but to study temporal characteristics of the coupling the dcMEG and related stimulation paradigms seem more appropriate. Often neurovascular coupling is studied through the combination of electroencephalography (EEG) and functional magnetic resonance imaging (fMRI) [9]. This combination again allows to record only fast neuronal responses and the EEG contains strong fMRI induced artifacts. 

An intermittent finger movement of 30-second duration was chosen as the paradigm to induce neurovascular responses here. The finger movement was monitored by recording the lower arm muscle EMG. This allows to employ the well-known coupling between EEG or MEG signals of the motor cortex and peripheral muscle activity [10] as a control parameter. Using independent component analysis (ICA) single unaveraged time series related to the finger movements were extracted from the multichannel dcMEG and trNIRS data. The frequently applied coherence cannot be estimated here in a meaningful way as 30 event repetitions in a 30-minute measurement session are not sufficient to obtain reliable spectral statistics. Therefore the coupling between the dcMEG, trNIRS, and EMG time series was analyzed using the cross-correlation function (CCF). The suitability of this approach for our experimental setting is assessed.

## 2. Material and Methods

### 2.1. Measurement Technique and Preprocessing

The measurement setup is similar to the setup combining modulated dcMEG with trNIRS as described in [4]. The trNIRS instrument operates at the wavelengths 690 nm, 803 nm, and 826 nm and is equipped with one source and four detector optodes. The detector optodes are arranged in a cross with the source in the center and a source-detector separation of 3 cm. In the present study, the logarithm of the relative count rate, that is, the change in attenuation at each wavelength with respect to a baseline interval, is used as NIRS parameter for the analysis. This allows the application of ICA at the wavelength level, which is not possible anymore after estimation of oxy- and deoxy-hemoglobin changes from the attenuation. With respect to the dcMEG, the present setup is improved employing the stationary, unmodulated dcMEG technique [3] with a sampling rate of 500 Hz. Both, dcMEG and trNIRS sensors, were approximately centered above the motor cortex contra-lateral to the finger movements. To control the subjects performance, the peripheral signals EMG, heartbeat, and respiration were measured in simultaneous recording tracks. The data set presented here is a representative example from a group study of six subjects. 

**Figure 1:**
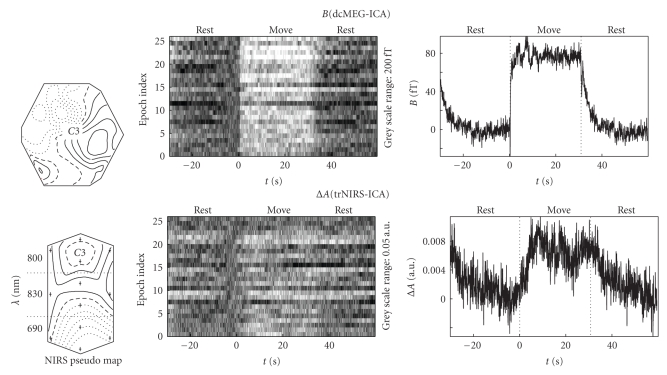
ICA components for *B* (dcMEG) and Δ*A*(trNIRS) due to finger movement. The component and its time series are characterized by the ICA map (left, see text for details of trNIRS map), the ST plot of the time series (middle), and the epoch average of the time series (right). In the averages the signal change between rest and move is clearly visible; in the ST plot it is more pronounced for *B*. The slower onset of Δ*A* compared to *B* at the beginning of the move window is obvious.

A highly structured finger movement pattern of the right hand was used as the stimulation paradigm. This movement pattern requires some practice before the measurement and ensures the attention of the subject during the experiment. The paradigm consists of a continuous sequence of finger contractions of the right hand: 2 ∗ thumb, 2 ∗ ring finger, 2 ∗ index finger, 2 ∗ middle finger, and 2 ∗ little finger. The two contractions of each finger should be performed within a second and the full sequence lasted typically 5 seconds. Naturally the individual speed was variable. Following an auditory cue, which was the prerecorded word “fast” chosen for its brevity and motivating appeal, the subjects had to start this structured finger movement. After 30 seconds of continuous movement another cue (“stop”) indicated the start of the 30 seconds rest period. This cycle was repeated 30 times resulting in a measurement duration of 30 minutes. The first and last two epochs (trials) from a measurement session were discarded, in order to eliminate edge effects due to highpass filtering of the raw dcMEG data at a frequency of 0.01 Hz, so that 26 of the 30 epochs remain (see Figure 1). In the following both the on/off cycles, that is, the intermittent finger movement and the rhythm of the finger movement within each 30 seconds period will be important.

To obtain the response due to the intermittent finger movement paradigm ICA was applied separately to dcMEG and trNIRS as demonstrated before in [7]. The ICA algorithm chosen was SOBI/TDSEP [11, 12], which is well suited to extract signals with a clear spectral structure as expected here due to the repetitive block design paradigm with 30 seconds of movement followed by 30 seconds of rest. The actual calculation is performed in the time domain as cross-covariances for a given delay *τ*_*k*_ between signal channels *x*_*i*_: *C*_*ij*_(*τ*_*k*_) = ∑*x*_*i*_(*t*)*x*_*j*_(*t* + *τ*_*k*_). A group of matrices {*C*(*τ*_*k*_)} is then diagonalised and the approximate diagonalising operator yields the independent components. 

The groups of component time series resulting from the ICA for the magnetic field *B*(*t*) and the attenuation Δ*A*(*t*) are searched each for a single time series with the highest correlation to the stimulation. These *B*(dcMEG-ICA)) and Δ*A*(trNIRS-ICA) time series and its associated component maps are then taken as the neuronal and vascular response to the finger movement. Only a single component was selected as all other components had a weak correlation with the stimulation sequence. Clearly it cannot be decided whether the single ICA component accounts for the whole motor response, but individual data sets with a good signal-to-noise ratio indicate this through the similarity between the ICA component and the standard average. At present the search is not automated, but the epoch averages of the ICA component time series are inspected manually. Although the ICA calculation is performed on nonaveraged raw data, the averaged ICA time series will be shown below. 

Two parameter options have to be chosen for the SOBI/TDSEP algorithm: the first is the bandwidth of the signals and the second is the set of delays {*τ*_*k*_}. Both parameters have to be chosen heuristically due to the lack of a theoretical foundation. In the MEG strong cortical background signals can be found in the *α*- and *β*-band starting around 8 Hz. It was found that ICA extracts a better movement-related response if the signal bandwidth is limited to 0.01–8 Hz excluding the background signals. The upper limit of 8 Hz has the additional advantage that then dcMEG and trNIRS have a similar upper band limit as the trNIRS is sampled at 20 Hz. The lower limit of 0.01 Hz is chosen to exclude monotonous signal drifts during the 30-minute measurement. Such monotonous signals are nonstationary and violate the ICA assumptions. Filtering as preprocessing is often performed implicitly by analog filters in the signal conditioning chain if ICA is applied to MEG data with a bandwidth of, for example, 1 to 100 Hz.

The set of delays {*τ*_*k*_} was optimized in a heuristic search using all data sets from the group study; that is, for a new set of delays SOBI/TDSEP was recalculated for all data sets in the group. This search was possible through a parallel implementation of the ICA algorithm running on a PC cluster (http://www.rocksclusters.org/) using a parallel computation interface (http://www.open-mpi.org/) and optimized linear algebra routines (http://math-atlas.sourceforge.net/). The final set of delays chosen for SOBI/TDSEP was {*τ*_*k*_} = {0.2, 0.4, 0.6,…, 60.0, 60.012, 60.024,…, 120.0} seconds. This set does not consist of evenly spaced delays, but it has a higher number of cross-covariance matrices at delays *τ*_*k*_ in the range of the 60 seconds stimulus repetition rate. This turned out to extract a better movement-related response.

From the measured EMG a rate (EMGR) was calculated by a process called amplitude demodulation. The full bandwidth EMG (sampling frequency 500 Hz, lowpass 250 Hz) was rectified and then input into a modified Paynter filter (readily available in http://sourceforge.net/projects/biosig/) with an 8 Hz lowpass characteristics. The resulting signal is the envelope of the oscillatory EMG signal. With respect to the subsequent cross-correlation analysis it was important that all signals had the same bandwidth. Therefore the heart rate derived from the ECG was filtered with the 8 Hz lowpass too.

### 2.2. Cross-Correlation Analysis

The CCF (e.g., [13]) is a tool to detect common periodicities between two time series, if the length of the time series is large compared to the oscillation period of interest. Given two time series *u*_*t*_ and *v*_*t*_ with *n* points their cross-correlation as a function of lag *τ* is given by

(1) $$\text{CC}{\text{F}}_{uv}\left(\tau \right)=\frac{1}{n\sqrt{\text{VAR}\left(u_{t}\right)\text{VAR}\left(v_{t+\tau }\right)}}\overset{t_{n}}{\underset{t=t_{0}}{\sum }}\left(u_{t}-{\overset{̅}{u}}_{t}\right)\left(v_{t+\tau }-{\overset{̅}{v}}_{t+\tau }\right),$$

where *τ* = 0,1,…, (*m* − 1) up to a maximum lag *m* ≪ *n* and VAR(*x*) is the sample variance. Due to the shifting of *v*_*t*_ by the lag **τ** the absolute positions of the cross-correlation maxima are related to phase shifts [13] for signals with similar basic periodicity. 

For the time series related to the intermittent finger movements two types of CCFs were investigated. The first was calculated using the complete unaveraged ICA time series of 26 minutes duration covering all rest/move sequences. This CCF probes for the coupling related to the transition between the two states, that is, fingers moving and at rest. The second was calculated for a new time series created by concatenation of the *move windows* with their mean values subtracted to minimize steps between the windows. This second type of CCF probes the coupling due to the rhythm of the finger movement. CCFs were calculated pairwise for a “signal triangle” consisting of *B*, Δ*A*, and EMGR.

## 3. Results

### 3.1. dcMEG, trNIRS, and EMGR Time Series

**Figure 2:**
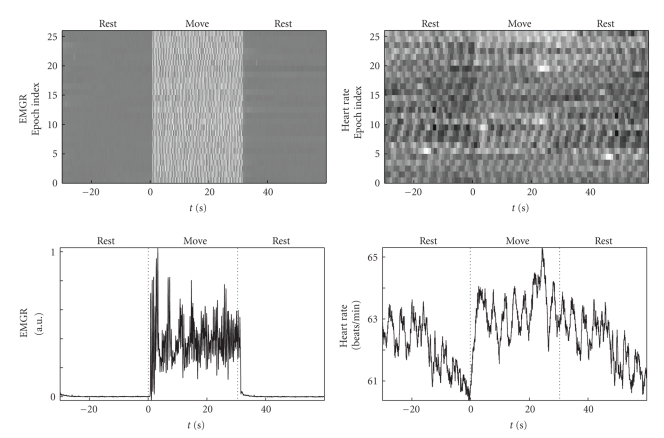
ST plots and resulting averages over the epochs of EMG rate (top) and heart rate (bottom). The ST plots show that the changes observed in the average are not due to outliers in a single epoch.

The continuous dcMEG and trNIRS time series extracted by ICA, *B*(dcMEG-ICA) and Δ*A*(trNIRS-ICA), are presented in Figure 1 using single trial (ST) plots. In an ST plot the continuous data are segmented into identical intervals and aligned at the time of the auditory start instruction. Epochs are presented in a stacked plot, where the amplitude value is coded as a shade of grey. This results in a two-dimensional grey-scale image of the complete continuous time series. A baseline correction is calculated in the 5-second window prior to finger movement onset. In Figures 1 and 2 the zero of the time axis corresponds to the auditory start instruction and the labels “rest” and “move” are added to emphasize the different regions. The dashed vertical lines in the average indicate the start and end of the “move” window.

ICA-extracted maps, ST plots, and averages of the finger movement-induced responses are shown for *B* and Δ*A* in Figure 1. For Δ*A* a pseudomap is used, which has a vertically elongated outline and dotted horizontal lines separating three wavelengths zones. In each zone the geometrical arrangement of the four detector optodes is indicated by the large dots. The interpolation is performed across the map and, therefore, the transitions across a dotted horizontal line have no meaning and each zone should be considered separately. The *B*-field map is a conventional field map displaying multichannel (scalar) magnetic field data. The motor cortex for right hand finger movements is typically associated with the EEG position C3 in the international 10–20 system for electrode placement and C3 is shown in the *B*-field map and the 800 nm part of the Δ*A* pseudomap in Figure 1. A source close to the center of the dcMEG sensor can be deduced from the dipolar structure of the *B*-field map. This is the expected result for position C3 and motor activity. The four-detector trNIRS setup covering roughly an area of 9 cm^2^ does not allow an accurate localization of the cortical trNIRS response, but the different sign of Δ*A* at 690 and 830 nm is a typical signature of cortical activity. This confirms that the positioning above the motor cortex was successful.

For *B* almost all epochs show a response during finger movement in the ST plots in Figure 1 and for Δ*A* the same holds despite a larger variability. Note that the grey-scale range in the ST plot for Δ*A* is five times larger than the resulting average value. This is a consequence of the large variability in the Δ*A* single responses, which is most likely due to physiological noise. The overall stable responses in the ST plots show that the time series extracted by ICA are correctly attributed to the intermittent finger movements. Most important is the immediate rise in the *B* signal (neuronal response) at finger movement onset contrasting with the much slower rise in the Δ*A* signal (vascular response).

The ST plots and associated epoch averages of the peripheral signals are shown in Figure 2. The sharp rise in the EMGR after *t* = 0 seconds both in the ST plot and the average shows the immediate start of finger movements. The heart rate shows a rapid increase at the beginning of the finger movement followed by oscillations around a constant value and a slow decrease after the end of finger movements. In the EMGR and the heart rate oscillations with a periodicity of 3 seconds to 4 seconds are visible in Figure 2. It is beyond the scope of this work to investigate this effect in detail, but preliminary cross-correlation results indicate coupling between heart rate and EMGR. 

### 3.2. Cross-Correlation

**Figure 3:**
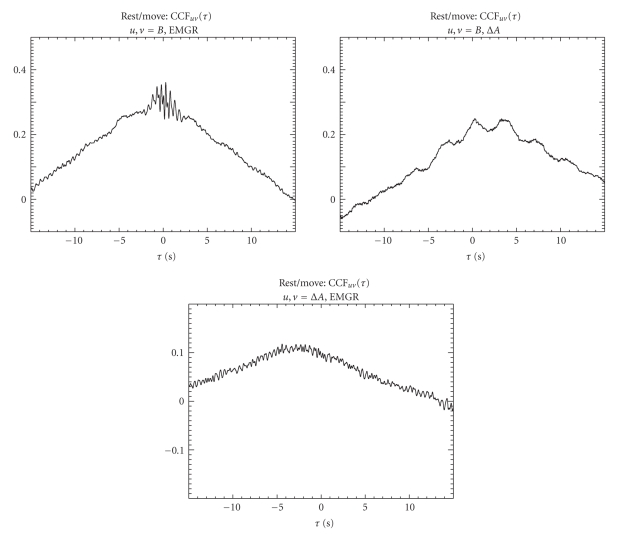
Pairwise CCFs of the measured modalities using the continuous time series, that is, including all rest/move cycles, for time lags from *τ* = −15 to 15 seconds. Note the different *y*-axis of the bottom figure. The highest correlations are reached for CCF(*B*, EMGR) and the curve is fairly symmetric with respect to zero lag (*τ* = 0 seconds). The correlations are slightly lower for CCF(*B*, Δ*A*) and the peak of the curve is shifted to positive *τ*. Low correlations result for CCF(Δ*A*, EMGR) and the peak of the curve is shifted to negative *τ*.

The pairwise CCFs of the full continuous time series covering all rest/move epochs are shown in Figure 3 for the “signal triangle” consisting of *B*(dcMEG-ICA), Δ*A*(trNIRS-ICA), and EMGR. For CCF(*B*, EMGR) the maximum correlation is reached around *τ* = 0 seconds with superimposed oscillations. For CCF(*B*, Δ*A*) the peak of the curve is shifted to (positive) *τ* ~ 2 seconds, which means that Δ*A* has a phase lag towards *B* as the order of the arguments is relevant for CCF(*t*) (see equation (1)). This phase shift is consistent with the data in Figure 1. The peak of the CCF(Δ*A*, EMGR) curve is shifted to negative *τ*, which is consistent with the immediate EMGR increase following finger movement onset as can be seen in Figure 2 and the much slower signal change in Δ*A* in Figure 1. The lower absolute correlation of CCF(Δ*A*, EMGR) compared to CCF(*B*, EMGR) shows that the overall signal shape differs more for CCF(Δ*A*, EMGR) again consistent with Figures 1 and 2. The peak shift to positive *τ* in CCF(*B*, Δ*A*) was seen before in a preliminary investigation [7].

**Figure 4:**
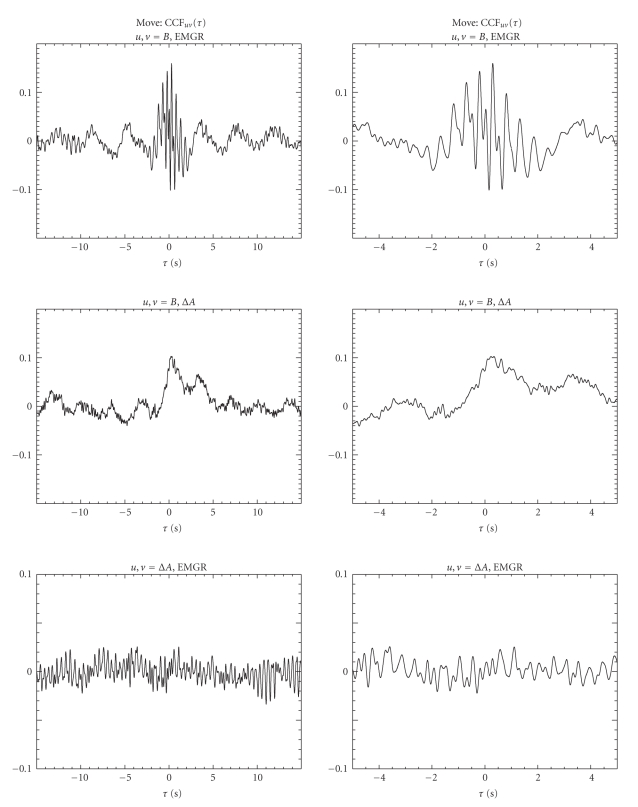
Pairwise CCFs for the time series consisting of the concatenated move windows. Two time lag ranges are displayed: *τ* = −15 to 15 seconds (left column) and *τ* = −5 to 5 seconds (right column). Note the different *y*-axis of the figures at the bottom. Two separate periodicities are observed only in CCF(*B*, EMGR).

In Figure 4 the CCFs of the time series consisting of the concatenated *move windows* are shown for the “signal triangle” for two different *τ* ranges. The CCF(*B*, EMGR) function in Figure 4 (top) shows rapid oscillations around *τ* = 0 seconds and slower oscillations with a period of 4 seconds throughout the **τ** range. Such clear oscillations are observed neither in CCF(*B*, Δ*A*) nor in CCF(Δ*A*, EMGR). The detailed plot of CCF(*B*, EMGR) around *τ* = 0 seconds (right column) shows that the rapid oscillations have a periodicity of 0.5 seconds. These rapid oscillations could be attributed to the coupling between neuronal signal and each individual finger contraction. This interpretation is consistent with the disappearance of the fast oscillations with increasing *τ*. The naturally somewhat irregular rhythm of individual finger movements for the duration of the 30-second move windows implies that only short range temporal correlations exist. For larger *τ* possibly the envelope of the finger contraction sequence is reflected in the CCF as the periodicity of 4 seconds is in the expected range. In CCF(*B*, Δ*A*) oscillations similar to the slow oscillations in CCF(*B*, EMGR) are observed, which could be interpreted as variations in vascular demand related to the envelope of the finger movements. The 4-second periodicity of the slow oscillations is not in contradiction to the time scale of neurovascular coupling [1–3]. The indirect coupling from EMGR to *B* and then to Δ*A* is apparently ineffective as CCF(Δ*A*, EMGR) is rather small and irregular. 

## 4. Conclusions

The synchronous triple measurement of dcMEG, trNIRS, and EMGR enabled a CCF analysis on continuous time series related to intermittent finger movements. The well-known difference in transition time for vascular and neuronal responses is reflected in a peak shift to positive *τ* for CCF(*B*(dcMEG-ICA), Δ*A*(trNIRS-ICA)). The value of the peak shift might serve as a parameter [13] to quantify the temporal characteristics of neurovascular coupling. A coupling on the basis of individual finger movements is likely between EMGR and *B* as their CCF shows oscillations at the finger movement frequency. This means that the unmodulated dcMEG with the increased bandwidth up to 8 Hz represents both slow and faster neuronal processes. The absence of apparent coupling between Δ*A* and EMGR indicates that the vascular response does not follow the individual finger movements. This is in agreement with the temporal characteristics of neurovascular coupling [1–3], that is, the peak shift in CCF(*B*, Δ*A*) here. The triple measurement in combination with the signal extraction by ICA and the CCF analysis allows a powerful coupling analysis at several time scales. Future work will try to incorporate spontaneous and induced fluctuations in the finger movement intensity to characterize the temporal behavior and the linearity of the coupling in a single experiment.
